# The Cytokine Release Inhibitory Drug CRID3 Targets ASC Oligomerisation in the NLRP3 and AIM2 Inflammasomes

**DOI:** 10.1371/journal.pone.0029539

**Published:** 2011-12-22

**Authors:** Rebecca C. Coll, Luke A. J. O'Neill

**Affiliations:** School of Biochemistry and Immunology, Trinity College Dublin, Dublin, Ireland; University of California Merced, United States of America

## Abstract

**Background:**

The Inflammasomes are multi-protein complexes that regulate caspase-1 activation and the production of the pro-inflammatory cytokine IL-1β. Previous studies identified a class of diarylsulfonylurea containing compounds called Cytokine Release Inhibitory Drugs (CRIDs) that inhibited the post-translational processing of IL-1β. Further work identified Glutathione S-Transferase Omega 1 (GSTO1) as a possible target of these CRIDs. This study aimed to investigate the mechanism of the inhibitory activity of the CRID CP-456,773 (termed CRID3) in light of recent advances in the area of inflammasome activation, and to clarify the potential role of GSTO1 in the regulation of IL-1β production.

**Methodology and Results:**

In murine bone marrow derived macrophages, CRID3 inhibited IL-1β secretion and caspase 1 processing in response to stimulation of NLRP3 and AIM2 but not NLRC4. CRID3 also prevented AIM2 dependent pyroptosis in contrast to the NLRP3 inhibitors glyburide and parthenolide, which do not inhibit AIM2 activation. Confocal microscopy and Western blotting assays indicated that CRID3 inhibited the formation of ASC complexes or ‘specks’ in response to NLRP3 and AIM2 stimulation. Co-immunoprecipitation assays show that GSTO1 interacted with ASC.

**Significance:**

These results identify CRID3 as a novel inhibitor of the NLRP3 and AIM2 inflammasomes and provide an insight into the mechanism of action of this small molecule. In addition GSTO1 may be a component of the inflammasome that is required for ASC complex formation.

## Introduction

The production of the pro-inflammatory cytokine interleukin (IL)-1β is a highly regulated process. An initial signal through the activation of pattern recognition receptors such as Toll-like receptors (TLRs) induces pro-IL-1β mRNA synthesis. Pro-IL-1β is biologically inactive and requires processing to generate the active 17 kilodalton (kDa) form that is secreted. Pro-IL-1β can be processed by caspase-1 which itself requires processing to form the active enzyme. The activation of caspase-1 is mediated by high molecular weight protein complexes termed inflammasomes [1], [2]. In addition to processing IL-1β and the related IL-1 family cytokine IL-18, caspase-1 also plays a role in unconventional protein secretion [3] and mediates a form of cell death called pyroptosis [4].

The Nod-like receptor protein NLRP3 forms the prototypical inflammasome by interacting with the adapter molecule apoptosis-associated speck-like protein containing a CARD (ASC) via its Pyrin domain (PYD). The caspase activation and recruitment domain (CARD) of ASC in turn binds the CARD domain of caspase-1 [4], [5]. NLRP3 can be activated in response to a highly diverse range of pathogen, environmental and endogenously derived molecules. Danger molecules such as ATP, pore forming toxins such as nigericin [6], particulates such as monosodium urate crystals [7] and fibrils such as islet amyloid polypeptide [8] are all sensed by NLRP3. NLRP3 is not directly activated but appears to sense an intermediate process or cellular perturbation caused by these molecules. These may include potassium efflux, the release of lysosomal proteases and the generation of reactive oxygen species [9]. NLRP3 protein expression levels are also a limiting step in inflammasome activation. NLRP3 thus requires induction or priming by TLR, NLR, IL-1 or TNFα stimulation [10].

Other NLR proteins such as NLRP1 and NLRC4 also form inflammasomes. NLRC4 senses bacterial flagellin and the rod protein from the type III secretion system apparatus of Gram-negative bacteria. It requires another NLR family member either NAIP5 or NAIP2 to detect its ligands [11], [12]. Absent in melanoma-2 (AIM2) is a non-NLR protein that is also capable of forming an inflammasome. AIM2 is a member of the PYHIN protein family that contain PYD domains and Hematopoietic expression, IFN-inducible, nuclear localisation (HIN) domains [13]. AIM2 recognises and directly binds cytosolic dsDNA via its HIN domain and recruits ASC to activate caspase-1. AIM2 is a broad sensor of dsDNA as it recognises viral, bacterial, mammalian and synthetic dsDNA [14], [15], [16], [17].

In a screen for inhibitors of IL-1β production a novel class of sulfonylurea containing compounds were identified. These so-called cytokine release inhibitory drugs or CRIDs (CP-424,174 and CP-412,245) inhibited the post-translational processing and secretion of IL-1β in response to LPS and ATP in human monocytes [18]. Further studies identified glutathione-S-transferase omega 1 (GSTO1) as a possible target for CRIDs [19].

The discovery of CRIDs predates the discovery of the inflammasomes. In this report we sought to characterise the inhibitory activity of the CRID CP-456,773 (termed CRID3) against multiple inflammasomes. We found that CRID3 inhibited both NLRP3 and AIM2 inflammasomes by preventing ASC oligomerisation. In addition GSTO1 was found to associate with ASC suggesting that it might play a role in inflammasome signalling and could indeed be a target of CRID3.

## Results

### CRID3 inhibits NLRP3 dependent IL-1β processing

**Figure 1:**
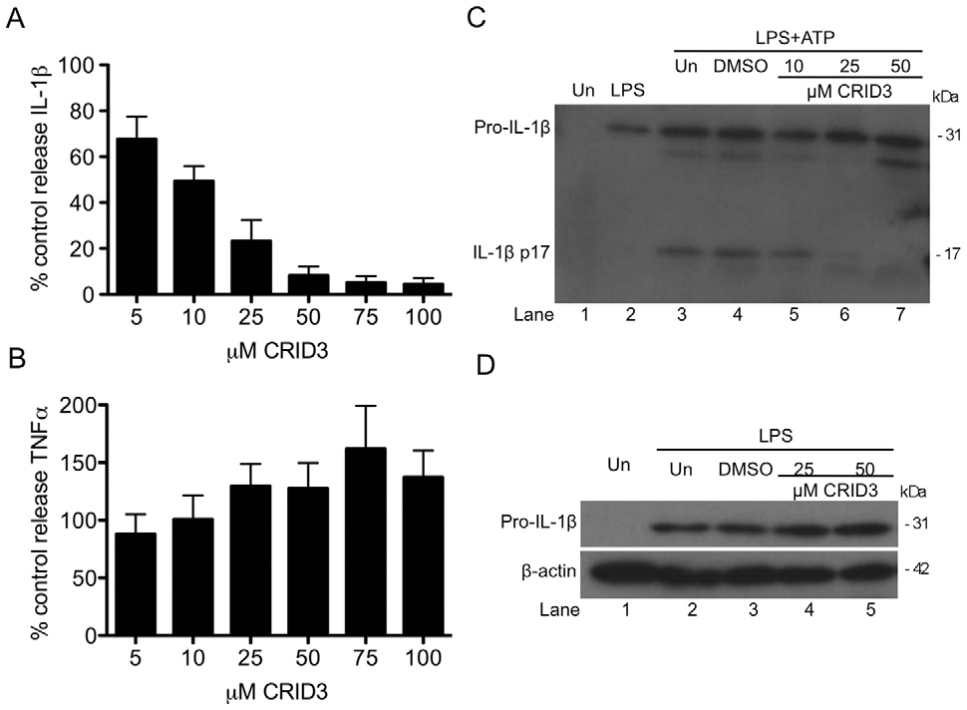
CRID3 inhibits LPS and ATP induced IL-1β processing. Primary BMDM were stimulated with 10 ng/ml LPS for 3 h, treated with CRID3 (5–100 µM) or DMSO in serum free media for 30 min followed by addition of 5 mM ATP for 1 h. Supernatants were analysed by ELISA for IL -1β (A), TNFα (B) or by Western blotting for pro and mature IL-1β (C). (A) and (B) Cytokine level is expressed as a percentage of that released from control treated cells, which ranged across all experiments from 521–1988 pg/ml for IL-1β and 104–211 pg/ml for TNFα. Data are expressed as mean ± S.E.M of five independent experiments each carried out in triplicate. (C) A representative blot from one of three independent experiments is shown. (D) BMDM were treated with DMSO or CRID3 (25–50 µM) for 30 min and were then stimulated with 10 ng/ml LPS for 3 h. Protein samples were analysed by Western blotting using anti-IL-1β and anti-β-actin antibodies. A representative blot from one of two independent experiments is shown.

The effect of CRID3 on the NLRP3 inflammasome was examined in bone marrow derived macrophages (BMDM). As shown in Figure 1A treatment with CRID3 (5–100 µM) dose dependently inhibited the amount of IL-1β produced by BMDM stimulated with lipopolysaccharide (LPS) and the NLRP3 activator ATP, with 50 µM having an optimum effect. The inhibition of IL-1β secretion was specific as CRID3 did not inhibit TNFα secretion from these cells (Figure 1B). In Figure 1C it was confirmed by Western blotting that CRID3 prevented the ATP stimulated processing of pro-IL-1β to the mature 17 kilodalton (kDa) form, the effect being optimal at 50 µM (compare lanes 5–7 to lane 3). As shown in Figure 1D pre-treatment with CRID3 did not affect the induction of pro-IL-1β by LPS (compare lanes 4 and 5 to lane 3).

### The AIM2 inflammasome is inhibited by CRID3

**Figure 2:**
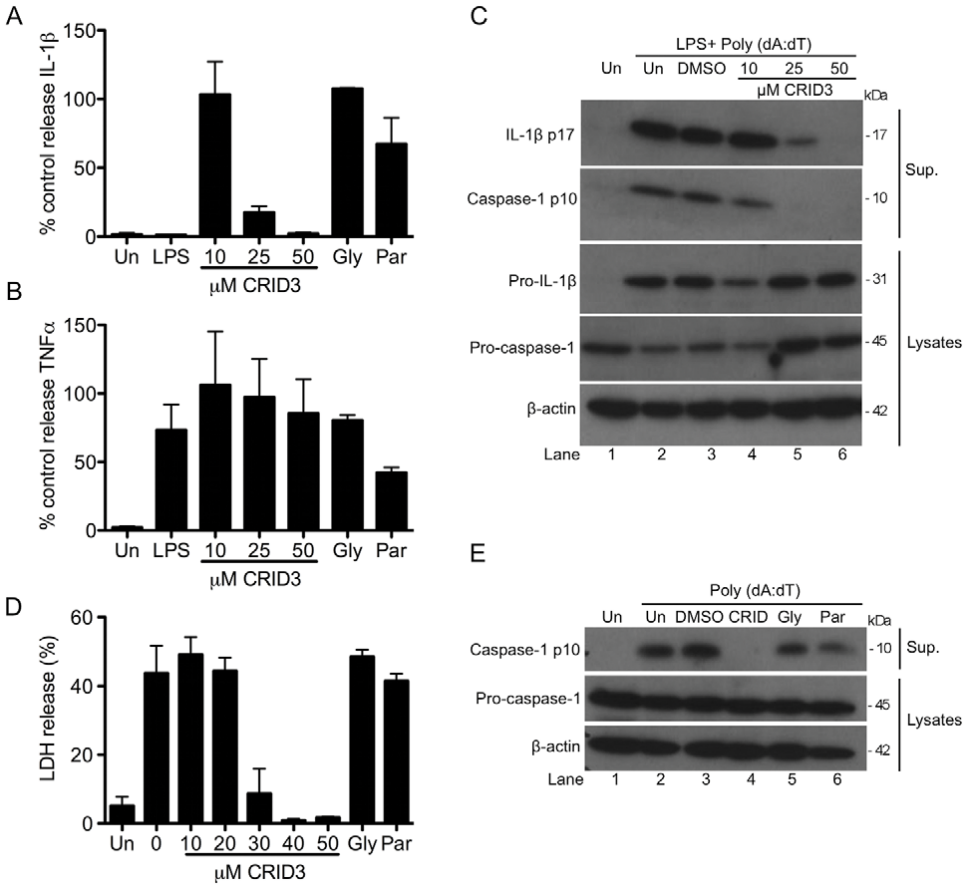
CRID3 inhibits the AIM2 inflammasome. Primary BMDM were stimulated with 10 ng/ml LPS for 3 h, treated with CRID3 (10–50 µM), 200 µM glyburide, 10 µM parthenolide or DMSO control in serum free media for 1 h followed by transfection of 1 µg/ml Poly (dA:dT) for 4 h (A) and (B) or overnight (C). Supernatants were analysed by ELISA for IL -1β (A) and TNFα (B). (A) and (B) Cytokine level is expressed as a percentage of that released from control treated cells, which ranged across all experiments from 518–2347 pg/ml for IL-1β and 397–1136 pg/ml for TNFα. Data are expressed as mean ± S.E.M of four independent experiments each carried out in triplicate. In (C) concentrated supernatants and cell lysates were analysed by Western blotting using anti-IL-1β, anti-caspase-1 and anti-β-actin antibodies. These results are representative of three independent experiments. (D) and (E) Primary BMDM were treated with CRID3 (10–50 µM), 200 µM glyburide,10 µM parthenolide or DMSO in serum free media for 1 h followed transfection of 1 µg/ml Poly (dA:dT) for 6 h. Supernatants were analysed using an LDH cytotoxicity assay. The data shown represent mean % LDH release ± S.D. from triplicate determinations and are representative of three independent experiments. In (E) Concentrated supernatants and cell lysates were analysed by Western blotting using anti-caspase-1 and anti-β-actin antibodies. These results are representative of four independent experiments.

As CRID3 was found to inhibit NLRP3 dependent responses we next tested its effect on the activation of other inflammasomes. Figure 2A shows that CRID3 inhibits the release of IL-1β from BMDM stimulated with LPS and the AIM2 activator synthetic dsDNA analog Poly (dA:dT) in a concentration dependent manner, optimum inhibition occurring at 50 µM. IL-1β secretion was not affected by treatment with the NLRP3 inhibitor glyburide [20] or parthenolide, which has also been shown to inhibit NLRP3 [21]. Glyburide and parthenolide both inhibited NLRP3 activation by LPS and ATP (data not shown). As shown in Figure 2B the secretion of TNFα from these cells was not significantly inhibited by CRID3 or glyburide, with parthenolide having a partial inhibitory effect. In Figure 2C treatment with CRID3 prevented the production of mature cleaved IL-1β and also prevented the processing of pro-caspase-1 as determined by detection of the p10 fragment in the supernatants. This effect was specific to the processing of IL-1β and caspase-1 as the levels of pro-IL-1β and pro-caspase-1 in the cell lysates were not decreased by treatment with CRID3 (lanes 5 and 6 in each case compared to lane 3).

In addition to cytokine processing AIM2 activation also leads to a caspase-1 dependent form of cell death known as pyroptosis. Figure 2D demonstrates that CRID3 prevents Poly (dA:dT) induced cell death, the effect being evident from 30 µM. Neither glyburide nor parthenolide inhibited this response. Figure 2E demonstrates that Poly (dA:dT) stimulated caspase-1 activation is also prevented in the presence of CRID3 (lane 4) but not glyburide (lane 5) or parthenolide (lane 6).

### CRID3 does not inhibit the NLRC4 inflammasome

**Figure 3:**
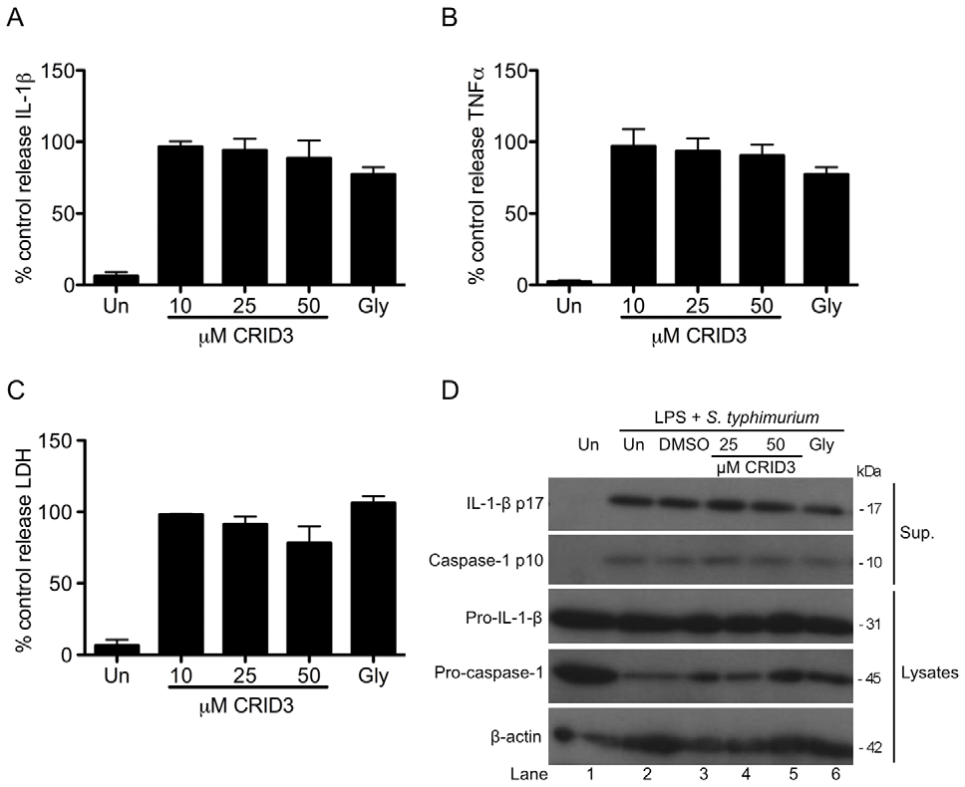
CRID3 and glyburide do not inhibit the NLRC4 inflammasome. Primary BMDM were stimulated with 10 ng/ml LPS for 3 h, treated with CRID3 (10–50 µM), 200 µM glyburide or DMSO in serum free media for 30 min followed by infection with *S. typhimurium* (M.O.I 20) for 2 h. Supernatants were analysed by ELISA for IL -1β (A) and TNFα (B) production or by LDH cytotoxicity assay (C). (A) and (B) cytokine level is expressed as a percentage of that released from control treated cells, which ranged across all experiments from 534–3013 pg/ml for IL-1β and 1990–2746 pg/ml for TNFα. (A), (B) and (C) data are expressed as mean ± S.E.M of three independent experiments each carried out in triplicate. In (D) concentrated supernatants and cell lysates were analysed by Western blotting using anti-IL-1β, anti-caspase-1 and anti-β-actin antibodies. These results are representative of three independent experiments.

The effect of CRID3 on the activation of the NLRC4 inflammasome was also tested. A previous report showed that in response to infection with *Salmonella typhimurium* at early time points IL-1β secretion and cell death are NLRC4 dependent [22]. In Figure 3 BMDM were primed with LPS prior to infection with *S. typhimurium*. Pre-treatment with CRID3 or glyburide did not significantly affect the levels of IL-1β (Figure 3A), TNFα (Figure 3B) or LDH (Figure 3C) released by cells. In agreement with the absence of an effect on IL-1β secretion or LDH release, pre-treatment with CRID3 or glyburide did not affect caspase-1 activation (Figure 3D). The levels of IL-1β p17 and caspase-1 p10 detected in the supernatants of cells stimulated with LPS and *S. typhimurium* was similar under every condition tested.

### CRID3 prevents NLRP3 and AIM2 dependent ASC oligomerisation

**Figure 4:**
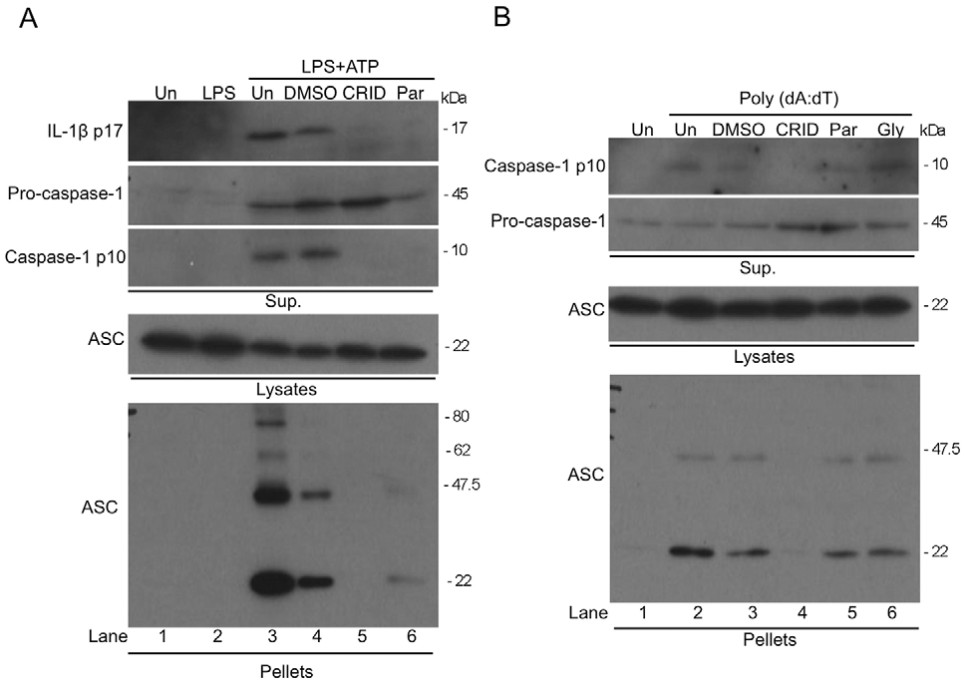
CRID3 prevents NLRP3 and AIM2 stimulated ASC oligomerisation. (A) I-BMDM were stimulated with 10 ng/ml LPS for 3 h, were treated with 50 µM CRID3, 10 µM parthenolide, DMSO or were left untreated in serum free media for 30 min followed by addition of 5 mM ATP for 1 h. (B) Cells were treated with with 50 µM CRID3, 200 µM glyburide,10 µM parthenolide, DMSO or were left untreated in serum free media for 30 min followed by transfection with 1 µg/ml Poly (dA:dT) for 5 h. Concentrated supernatants and cell lysates were analysed by Western blotting using anti-IL-1β and/or anti-caspase-1 antibodies. The cell lysates were centrifuged at 330 x g for 10 min at 4**°**C. The pellets were washed and resuspended in PBS and then cross-linked by incubation with DSS for 30 min. The cross-linked pellets and cell lysates were analysed by Western blotting with anti-ASC and anti-caspase-1 antibodies. The data shown are representative of two (A) and three (B) independent experiments.

The activation of NLRP3 and AIM2 results in the recruitment of ASC and the formation of large molecular weight oligomers that in turn activate caspase-1. Figure 4A demonstrates that stimulation of immortalized BMDM (I-BMDM) with LPS and ATP caused the formation of ASC dimers and higher order oligomers, which are detected in the cell pellet by Western blotting (bottom panel, compare lanes 2 and 3). However, the pre-treatment of cells with CRID3 and parthenolide before stimulation with ATP prevented ASC oligomerisation (bottom panel, lanes 5 and 6), the production of IL-1β (top panel lanes 5 and 6) caspase-1 activation (third panel, lanes 5 and 6). CRID3 and parthenolide did not affect ASC expression, as seen in the cell lysates (fourth panel). As shown in Figure 4B transfection of I-BMDM with Poly (dA:dT) also resulted in the appearance of ASC dimers in cell pellets (bottom panel, lane 2). CRID3 inhibited the activation of caspase-1 (top panel, lane 4) and the formation of ASC oligomers (bottom panel, lane 4) whereas parthenolide and glyburide had no effect on either response (top and bottom panels, lanes 5 and 6).

**Figure 5:**
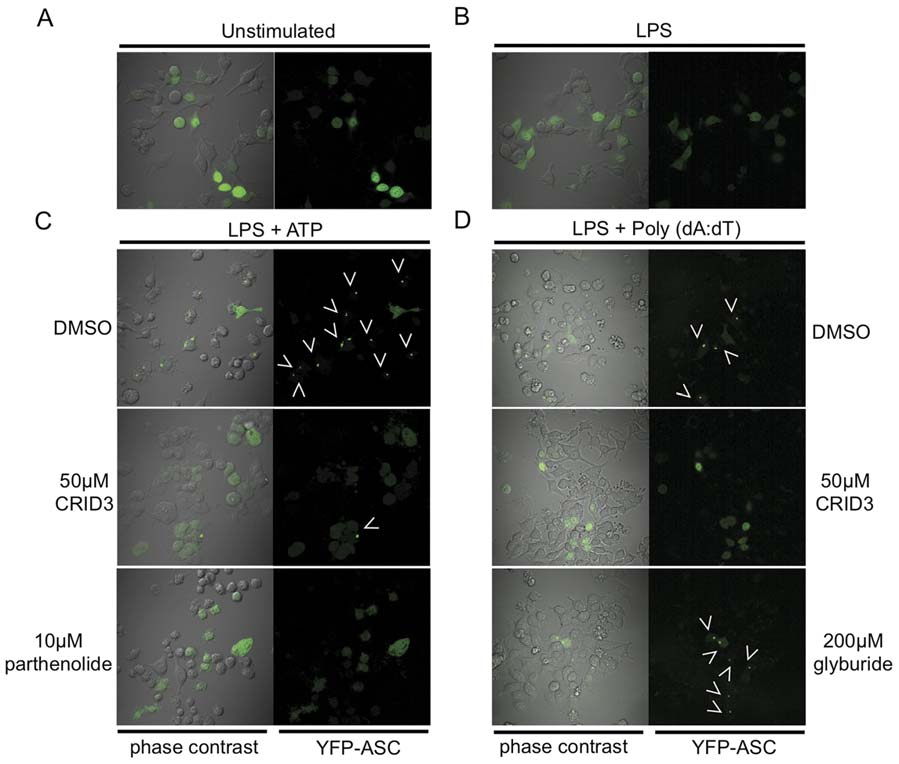
CRID3 inhibits NLRP3 and AIM2 stimulated ASC speck formation. (A) ASC-YFP cells were left unstimulated, or were (B) stimulated with 10 ng/ml LPS for 3 h. (C) Cells were stimulated with 10 ng/ml LPS for 3 h, treated with 50 µM CRID3 10 µM parthenolide or DMSO in serum free media for 30 min followed by addition of 5 mM ATP for 1 h. In (D) cells were stimulated with 10 ng/ml LPS for 3 h, treated with 50 µM CRID3, 200 µM glyburide or DMSO in serum free media for 40 min followed by transfection of 1 µg/ml Poly (dA:dT) for 2 h. Cells were viewed by live cell imaging, at least four different images were taken of each dish of which a representative image is shown. These results are representative of three independent experiments.

An additional assay was also employed to examine the activation of ASC oligomerisation by NLRP3 and AIM2. Murine immortalized macrophages that stably express ASC-YFP were used to analyse the formation of ASC oligomers or ‘specks’. As shown in Figure 5A in unstimulated cells or cells treated with LPS alone (Figure 5B) the YFP-ASC is diffuse throughout the cytosol of the cell, but in response to stimulation with LPS and ATP (Figure 5C, first row) or Poly (dA:dT) (Figure 5D, first row) the YFP-ASC condenses into a large, bright, speck. Arrowheads are included to indicate typical specks. CRID3 at 50 µM and 10 µM parthenolide prevented the formation of specks in response to stimulation with LPS and ATP (Figure 5C, second and third rows). As shown in Figure 5D CRID3 (50 µM) also inhibited speck formation in cells stimulated with LPS and Poly (dA:dT) (Figure 5dD second row), whereas glyburide had no effect (Figure 5D, third row).

### GSTO1 interacts with ASC

**Figure 6:**
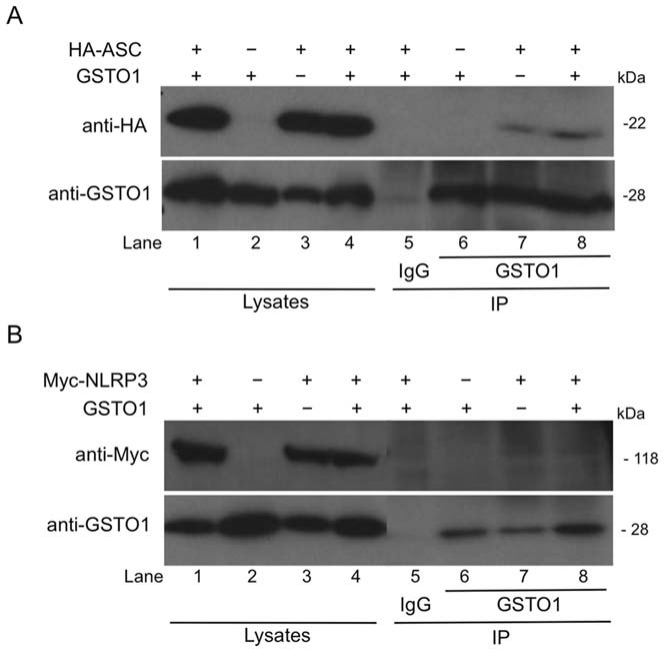
ASC but not NLRP3 immunoprecipitates with GSTO1. HEK-293T cells were transfected with HA-ASC, Myc-NLRP3, GSTO1 or empty vector plasmids as indicated. 48 hours post transfection cells were lysed in low stringency lysis buffer. 50 µl of lysate was kept for analysis while the remaining lysates were pre-cleared using 10 µl Protein A/G beads for 30 min. The lysates were then incubated with anti-GSTO1 antibody or rabbit IgG coupled to protein A/G beads for 3 h at 4°C. Whole cell lysates and immunoprecipitation samples were analysed by Western blotting using anti-Myc, anti-HA or anti-GSTO1 antibodies. The results presented are representative of two (B) and three (A) independent experiments.

Previous investigations into the target of the CRID compounds had identified GSTO1 as the primary candidate for their target of action [19]. As CRID3 inhibited both NLRP3 and AIM2 dependent responses it was investigated whether GSTO1 could interact with components of the inflammasome. As shown in Figure 6A ASC immunoprecipitated together with both endogenous lane 7) and overexpressed GSTO1 (lane 8) in HEK293T cells. Figure 6B shows that no interaction between NLRP3 and GSTO1 could be detected (lanes 7 and 8).

## Discussion

This study has identified CRID3 as an inhibitor of the NLRP3 and AIM2 inflammasomes but not the NLRC4 inflammasome. CRID3 inhibited ASC oligomerisation. A previously identified target of CRID3, GSTO1 was shown to interact with ASC suggesting that it may be required for ASC function in the inflammasome.

CRID3 dose dependently prevented IL-1β but not TNFα secretion from BMDM stimulated with LPS and ATP. This result is in line with previous studies on the effects of related CRID compounds [18], [19]. The decrease in IL-1β secretion was due to inhibition of IL-1β processing as CRID3 did not have an effect on pro-IL-1β induction. CRID3 was also found to inhibit the AIM2 inflammasome, preventing IL-1β secretion, caspase-1 activation and cell death in response to AIM2 activation by Poly (dA:dT).

The other NLRP3 inhibitors glyburide and parthenolide had no significant effect on AIM2 mediated responses pointing to specificity in their actions. Juliana et al. [21] have shown that parthenolide directly inhibits caspase-1 by alkylation of certain cysteine residues. Our results disagree with this assertion as we did not find that parthenolide inhibited caspase-1 in response to AIM2 stimulation. However, the previous study [21] did not test AIM2 activation so perhaps parthenolide only inhibits caspase-1 in response to NLRP3 or NLRC4 activation. Importantly CRID3 did not affect NLRC4 mediated caspase-1 activation and IL-1β secretion in response to *S. typhimurium* infection.

It was possible that CRID3 was targeting ASC, since ASC is required for the function of the NLRP3 and AIM2 inflammasomes but has a lesser role in the NLRC4 inflammasome. In both the Western blotting and ASC-YFP fluorescence microscopy assays it was found that pre-treatment with CRID3 prevented ASC oligomerisation in response to stimulation with LPS and ATP or Poly (dA:dT). In agreement with the effects seen previously on IL-1β secretion and caspase-1 activation glyburide and parthenolide did not inhibit AIM2 dependent ASC oligomerisation.

ASC is indispensible for both NLRP3 and AIM2 inflammasome formation. As CRID3 prevents ASC oligomerisation in response to activation of both these molecules this would suggest that CRID3 acts directly or at the level of ASC in these complexes. The role of ASC in the NLRC4 inflammasome is less clear. The caspase activation and recruitment domain (CARD) of NLRC4 can interact directly with caspase-1 but ASC facilitates more efficient IL-1β secretion [1], [23]. ASC does not play a role in NLRC4 induced cell death as in vivo studies showed NLRC4 mediated pyroptosis, which is critical for *S. typhimurium* clearance was independent of ASC [24]. CRID3 did not prevent *S. typhimurium* induced cell death and thus does not inhibit NLRC4. There may be a partial role for ASC in IL-1β induction in response to NLRC4, since at early time points this response is impaired in ASC deficient macrophages [22]. However we found that CP- CRID3 had no effect on NLRC4 dependent IL-1β secretion. This may be because ASC was not involved in the particular conditions we tested or because CRID3 specifically inhibits ASC on the NLRP3 and AIM2 pathways. From our ASC assays however, it is clear that CRID3 can inhibit ASC activation by NLRP3 and AIM2.

A previous study using affinity labelling and affinity binding chromatography techniques had identified GSTO1 as the target of CRID compounds [19]. GSTO are the most recently identified and characterised class of cytosolic GSTs. GSTO1 is an unusual GST as it contains an active site cysteine residue and displays little activity with typical GST substrates. It does however have thiol transferase activity that is characteristic of glutaredoxins [25]. An initial possible mechanism of CRID3 inhibition could have been through directly preventing caspase-1 activation. A previous study has shown that caspase-1 could be inhibited by glutathionylation of Cys397 and Cys362 during conditions of oxidative stress [26]. Thus it was possible that CRID3 could deglutathionylate caspase-1. However, as CRID3 failed to inhibit NLRC4 activated caspase-1 activation this suggested that CRID3 was not directly affecting caspase-1. It has been suggested previously that related CRID compounds were not direct caspase-1 inhibitors although the data was not shown in the report [18]. The physiological role of GSTO1 remains ill-defined although some studies have implicated a role in drug resistance and the oxidative stress response [27], [28]. Polymorphisms in GSTO1 were also shown to affect the risk of developing sporadic Alzheimer's disease [29].

We speculated that GSTO1 might interact with components of the inflammasome. Co-immunoprecipitation experiments indicated that GSTO1 did not interact with NLRP3 but did interact with ASC. GSTO1 is therefore a novel component of the NLRP3 and AIM2 inflammasomes. How GSTO1 affects ASC is open to speculation. As mentioned previously caspase-1 was shown to be negatively regulated by glutathionylation [26], ASC may also subject to this post-translational modification. There is an emerging literature indicating that reversible glutathionylation of proteins is a form of signal transduction analogous to protein phosphorylation or ubiquitination.

Glutathionylation has been implicated in the regulation of TNFα induced apoptosis through reversible glutathionylation of caspase-3 [30]. While the glutathionylation of endothelial nitric oxide synthase (eNOS) has been shown to regulate its function in vasodilation [31]. There are also examples of GSTs as regulators of cytokine signalling. GSTP regulates TNFα signalling by associating with tumour-necrosis factor receptor-associated factor 2 (TRAF2) and blocking the TRAF2 interaction with apoptosis signal regulating kinase 1 (ASK1). This activity of GSTP was independent of its glutathione conjugating activity [32]. Perhaps GSTO1 regulates the interaction of ASC with NLRP3 or AIM2 or with other ASC molecules, with CRID3 interfering with this interaction, preventing formation of the inflammasome protein complex.

There is significant evidence that ASC regulates innate immune responses separate to its role in the inflammasome. Studies on ASC deficient mice have shown that ASC is required for the induction of humoral immunity after vaccination with the MF59 adjuvant. ASC deficient mice had significantly reduced antigen specific IgG responses, whereas caspase-1 and NLRP3 deficient mice had normal responses [33]. In contrast to caspase-1 and NLRP3 deficient mice, ASC deficient mice were completely protected from the development of arthritis in a collagen induced arthritis study. This was due to defective T cell activation by ASC deficient dendritic cells [34]. ASC deficient mice were also more protected than caspase-1 deficient mice from a mouse model of multiple sclerosis (experimental autoimmune encephalitis or EAE) [35]. An inhibitor of ASC such as CRID3 could therefore be an effective treatment for these diseases. A recent study has also defined an inflammasome independent role for ASC in the post-transcriptional regulation of the guanine nucleotide exchange factor Dock2. ASC deficient dendritic cells had very low Dock2 expression, which led to decreased Rac dependent actin polymerization and consequently impaired antigen uptake and chemotaxis [36]. As GSTO1 interacted with ASC but not NLRP3 it is possible that GSTO1 may also influence these non-inflammasome related functions of ASC. Further studies on the mechanism of ASC function in these settings may establish if GSTO1 is required specifically or ubiquitously for ASC mediated immune responses.

Given the role of IL-1β in diseases such as type 2 diabetes [8], gout [7] and osteoarthritis [37], which all involve NLRP3, a small molecule such as CRID3 could have promise therapeutically and be an alternative to IL-1β biologics such as the anti-IL-1β antibody Canakinumab and the IL-1 Trap Rilonacept. Future work aims to elucidate the precise molecular target for CRID3.

## Materials and Methods

### Materials

Ultrapure rough LPS (from E. coli, serotype EH100) was purchased from Alexis. Poly(deoxyadenylic-thymidylic) acid sodium salt (Poly dA:dT) and adenosine 5′-triphosphate disodium salt hydrate (ATP) were purchased from Sigma-Aldrich. *S. typhimurium* UK-1 strain was obtained from Dr. Sinead Corr (Trinity College Dublin, Ireland). Glyburide was obtained from Sigma-Aldrich. Parthenolide was purchased from Enzo Life Sciences. CP-456,773 (CRID3) was from Amgen Inc., Thousand Oaks, CA, USA. GeneJuice® was purchased from Novagen. Lipofectamine 2000™ was from Invitrogen. Protein A/G Plus agarose immunoprecipitation reagent was from Santa Cruz Biotechnology. StrataClean**™** resin was from Agilent Technologies. TNF-α and IL-1β ELISA Duosets were purchased from R&D Systems. CytoTox96® non-radioactive cytotoxicity assay was from Promega.

Human GSTO1 pcDNA3.1 was a gift from Prof. Philip Board, John Curtin School of medical research, Australian National University, Australia. The pCI ASC-HA expression vector was a gift from Dr. Kate Fitzgerald, University of Massachusetts, USA. Empty vector pcDNA 3.1 myc-His was from Invitrogen. Myc-caspase-1 and myc-NLRP3 were gifts from Prof. Dan Kastner, National Institute of Arthritis and Musculoskeletal and Skin Diseases, Bethesda, Maryland, USA. ASC antibody (AL177) was from Enzo Life Sciences. Murine caspase-1 p10 (M-20) and c-Myc (9E10) antibodies were obtained from Santa Cruz Biotechnology. HA.11 antibody was from Covance. Murine IL-1β antibody was from R&D Systems. Rabbit antiserum raised against recombinant hGSTO1-1 was a gift from Prof. Philip Board, John Curtin School of medical research, Australian National University, Australia. β-actin, and anti-rabbit IgG were obtained from Sigma-Aldrich.

### Cell culture

The immortalised BMDM were a kind gift from Prof. Douglas Golenbock, University of Massachusetts, USA [38]. Stably transfected ASC-FP macrophages were a kind gift from Dr. Eicke Latz, University of Massachusetts, USA [39]. The human embryonic kidney T cell line (HEK-293T) was purchased from the European Cell Culture Collection. HEK293T, ASC-FP and I-BMDM cell lines were cultured in Dulbecco's modified Eagle's medium (DMEM) supplemented with 10% fetal calf serum (FCS) and 1% penicillin/streptomycin (v/v). Bone marrow from C57BL/6 mice was differentiated for 7 days in DMEM supplemented with 10% fetal calf serum (FCS), 1% penicillin/streptomycin (v/v) and M-CSF (20% (v/v) L929 mouse fibroblast supernatant). For experiments the differentiated BMDM were seeded at 0.25−1×10^6^/ml.

### Enzyme-linked immunosorbent assay

For cytokine measurements, BMDM were stimulated in triplicate as indicated. Supernatants were removed and analysed for IL-1β and TNF-α using enzyme-linked immunosorbent assay (ELISA) kits according to the manufacturer's instructions (R&D Systems).

### Western blotting

For Western blotting experiments BMDM were seeded in 12 or 6 well plates at 0.5−1×10^6^/ml. Cell lysates were prepared by direct lysis in 5X Laemmli sample buffer. The protein content of supernatants was concentrated using StrataClean™ resin according to the manufacturer's instructions. The protein samples were resolved on 12% or 15% SDS-PAGE gels and transferred onto polyvinylidene diflouride (PVDF) membrane using a wet transfer system. Membranes were blocked in 5% (w/v) dried milk in TBS-T (50 mM Tris/HCL, pH 7.6, 150 mM NaCl and 0.1% (v/v) Tween-20) for an hour at room temperature (RT). The membranes were incubated with primary antibody diluted 1 in 1000 in 5% (w/v) dried milk in TBS-T. The membranes were then incubated with the appropriate horse radish peroxidise conjugated secondary antibody diluted 1 in 2000 in 5% (w/v) dried milk in TBS-T for an hour before being developed by enhanced chemiluminescence (ECL) according to the manufacturer's instructions (Cell Signaling Technology, Inc.). Some membranes were stripped using Restore™ PLUS western blot stripping buffer according to the manufacturer's instructions (Thermo Fisher Scientific Inc.) before being re-probed.

### Cytotoxicity assay

A CytoTox96® non-radioactive cytotoxicity assay kit was used according to the manufacturer's instructions (Promega) to determine lactate dehydrogenase (LDH) release from cells.

### ASC complex isolation

2×10^6^ I-BMDM cells were seeded in each well of a 6 well plate. The following day cells were stimulated with 10 ng/ml LPS for 3 hours before treatment with the indicated inhibitors or controls for 30 mins followed by the addition of 5 mM ATP for 1 hour or cells were pre-treated with inhibitors for 30 mins followed by transfection of 1 µg/ml Poly (dA:dT) for 5 hrs. The supernatants were removed, cells were rinsed in ice-cold PBS and 500 µl ice-cold buffer (20 mM Hepes-KOH, pH 7.5, 150 mM KCL, 1% NP-40 0.1 mM PMSF, 1 µg/ml leupeptin, 11.5 µg/ml aprotinin and 1 mM sodium orthovanadate) was added. The cells were removed using a cell scraper and transferred to microcentrifuge tubes. Cells were lysed by shearing 10X through a 21 gauge needle. 50 µl of lysate was removed for Western blot analysis. The lysates were centrifuged at 330 x g for 10 min at 4°C. The pellets were washed twice in 1 ml ice-cold PBS (centrifuged 330 x g for 3 min at 4°C) and resuspended in 500 µl PBS. 2 mm disuccinimydyl suberate (DSS) (from a fresh 100 mM stock prepared from DSS equilibrated to RT and made up in dry DMSO) was added to the re-suspended pellets, which were incubated at RT for 30 min with rotation. The samples were then centrifuged at 330 x g for 10 min at 4°C. The supernatant was removed and the cross-linked pellets were resuspended in 30 µl Laemmli sample buffer. The samples were boiled for 5 min at 99°C and analysed by Western blotting.

### Confocal microscopy

ASC-FP cells were seeded at 2×10^5^/ml the day prior to use in experiments on 35 mm glass bottom culture dishes. Cells were stimulated with 10 ng/ml LPS for 3 hours before treatment with the indicated inhibitors or controls for 30–40 mins followed by the addition of 5 mM ATP for 1 hour or transfection of 1 µg/ml Poly (dA:dT) for 2 hrs. Imaging was performed on an Olympus FluoView™ FV1000 Microscope equipped with a temperature and CO_2_ controlled chamber.

### Co-immunoprecipitation assay

HEK293T cells were seeded at 2×10^5^ per ml. 24 hours later cells were transfected with a total of 8 µg of the indicated plasmids using GeneJuice®. Cells were cultured for 36–48 hours prior to harvesting in low stringency lysis buffer (50 mM Hepes pH 7.5, 100 mM NaCl, 1 mM ethylenediaminetetraacetic acid (EDTA), 10% glycerol, 0.5% Nonidet P40 (NP-40), 1 mM phenylmethylsulphonyl fluoride (PMSF), 1 µg/ml leupeptin, 11.5 µg/ml aprotinin and 1 mM sodium orthovanadate] on ice for 15 min. Lysates were centrifuged at 1610 x g for 10 min at 4°C. Lysates were pre-cleared for 30 min with 10 µl Protein A/G Plus agarose beads. Prior to incubation with lysates 1 µg of the relevant antibodies was incubated with 25 µl Protein A/G Plus agarose beads overnight. The lysates were then incubated with pre-coupled antibodies/beads for 4 hrs rotating at 4°C. The lysates and beads were centrifuged at 80 x g for 3 min at 4°C, the supernatant was removed and the beads were washed 3 times in 1 ml of low stringency lysis buffer. The immune complexes were eluted by the addition of 35 µl Laemmli sample buffer, and analysed by SDS-PAGE and Western blotting.
