# Correction: The Cytokine Release Inhibitory Drug CRID3 Targets ASC Oligomerisation in the NLRP3 and AIM2 Inflammasomes

**DOI:** 10.1371/annotation/9f221489-155d-4978-a36d-30c51853e438

**Published:** 2013-02-27

**Authors:** Rebecca C. Coll, Luke A. J. O'Neill

In this article we reported that a compound provided by Amgen, termed CRID3, could inhibit both the NLRP3 and AIM2 inflammasomes. However, subsequent analysis of the Amgen compound by liquid chromatography-mass spectrometry (LC-MS), tandem mass spectrometry (MS-MS) and NMR (nuclear magnetic resonance) (see files attached to this Correction) revealed that the compound is a different structure, namely 1-(5-carboxy-2-{3-[4-(3-yclohexylpropoxy)phenyl]propoxy}benzoyl)piperidine-4-carboxylic acid as described by Härter, M. et al. U.S Patent 7,498,460, 2009.

This compound was developed at Bayer Pharmaceuticals and is a cysteinyl leukotriene receptor antagonist. CRID3 is an inhibitor of NLRP3 inflammasome activation with an IC50 of approx. 10nM, however, it does not inhibit AIM2 inflammasome activation.

The mass spectrometry and NMR analysis undertaken to characterize the compound, and which are included in this Correction, were carried out by Dr. Avril Robertson and Dr. Mark Butler in Prof. Matt Cooper's laboratory at the Institute for Molecular Bioscience, The University of Queensland, Australia. We would therefore like to revise the authors' list to the following:

Rebecca C. Coll, Avril A. B. Robertson, Mark S. Butler, Matthew A.Cooper, Luke A. J. O'Neill

Additional information supplied here. The first file is a summary with the details of the subsequent files:

[^]

[^]

[^]

[^]

[^]

[^]

[^]

[^]

[^]

[^]

