# Correction: The Cytokine Release Inhibitory Drug CRID3 Targets ASC Oligomerisation in the NLRP3 and AIM2 Inflammasomes

**DOI:** 10.1371/annotation/78d328b9-2c8c-4978-84b1-7e6a0b12ada1

**Published:** 2013-07-09

**Authors:** Rebecca C. Coll, Luke A. J. O'Neill

Drs. Avril Robertson and Mark Butler, and Prof. Matthew Cooper were not included in the author byline. They should be listed as the second, third and fourth authors and affiliated with the Institute of Molecular Bioscience, University of Queensland, Brisbane, Australia. The correct citation is: Coll RC, Robertson AAB, Butler M, Cooper M, O'Neill LAJ (2011) The Cytokine Release Inhibitory Drug CRID3 Targets ASC Oligomerisation in the NLRP3 and AIM2 Inflammasomes. PLoS ONE 6(12): e29539. doi:10.1371/journal.pone.0029539. The following sentence should be inserted in Contributions: "Mass spectrometry and NMR analysis: AABR, MB, MC." 

