# Supplementary figures and images for: Correction: The Cytokine Release Inhibitory Drug CRID3 Targets ASC Oligomerisation in the NLRP3 and AIM2 Inflammasomes
Source: PLoS One. 2013 Feb 27;8(2):10.1371/annotation/9f221489-155d-4978-a36d-30c51853e438. doi: 10.1371/annotation/9f221489-155d-4978-a36d-30c51853e438 (PMC3586581; doi:10.1371/annotation/9f221489-155d-4978-a36d-30c51853e438)

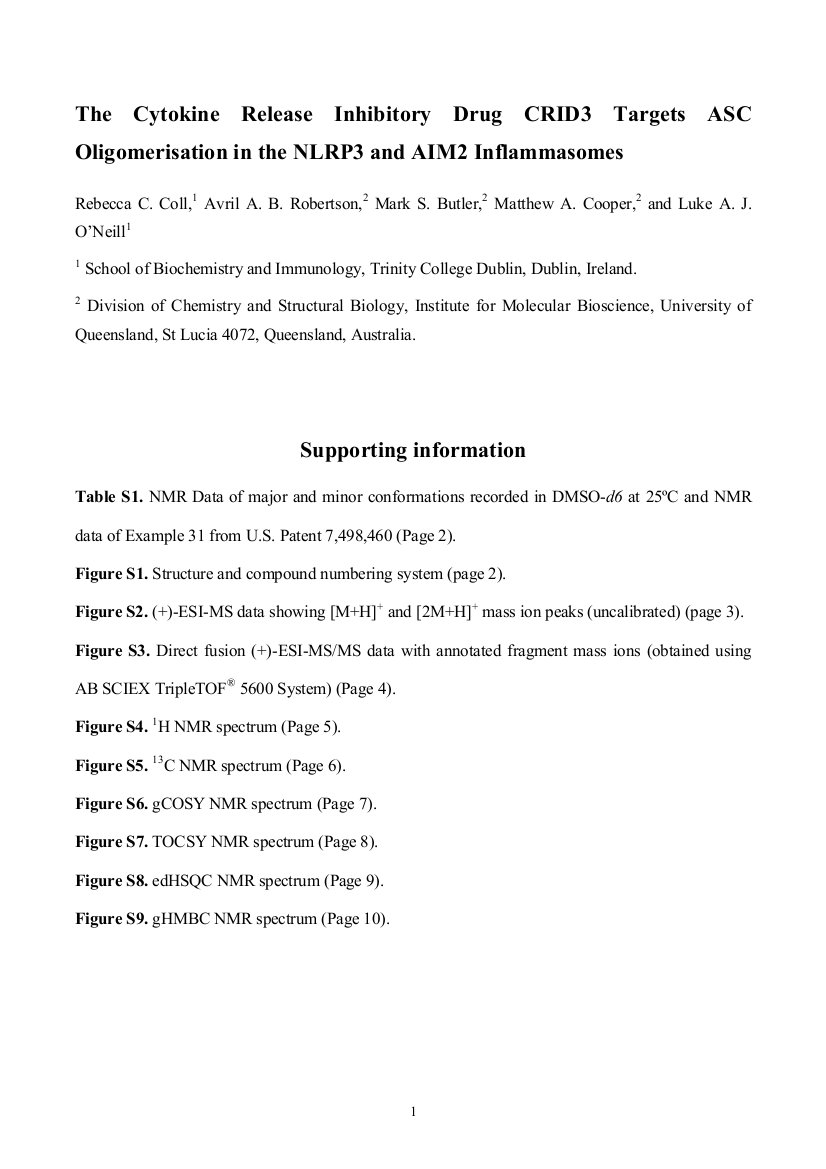

Supplement: Supplementary file 1 [file pone.9f221489-155d-4978-a36d-30c51853e438.s001.tif]

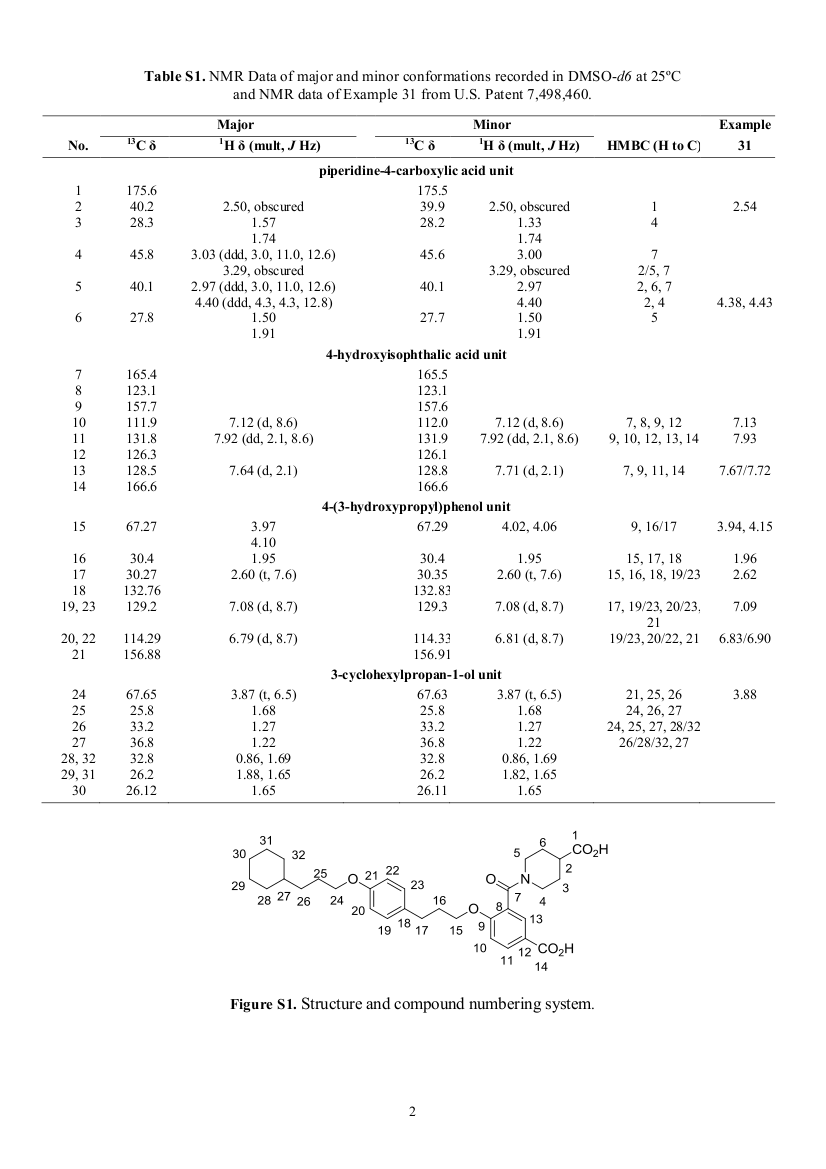

Supplement: Supplementary file 2 [file pone.9f221489-155d-4978-a36d-30c51853e438.s002.tif]

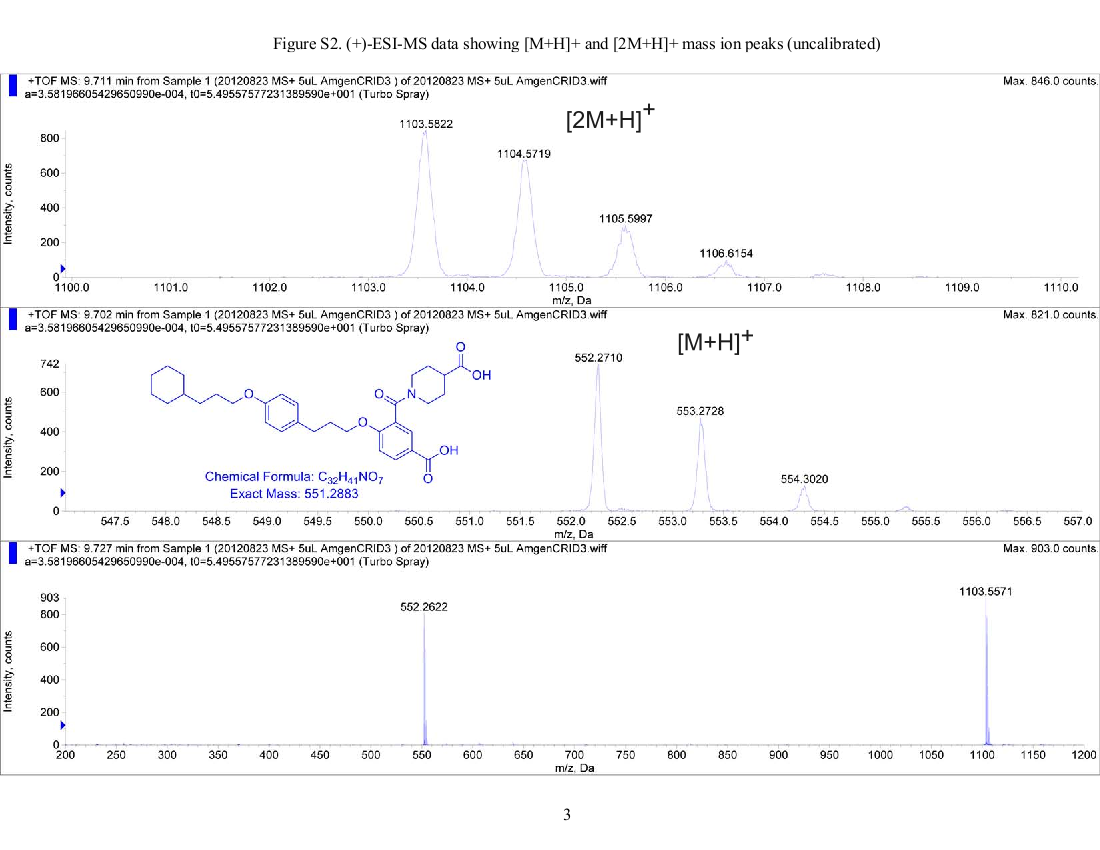

Supplement: Supplementary file 3 [file pone.9f221489-155d-4978-a36d-30c51853e438.s003.tif]

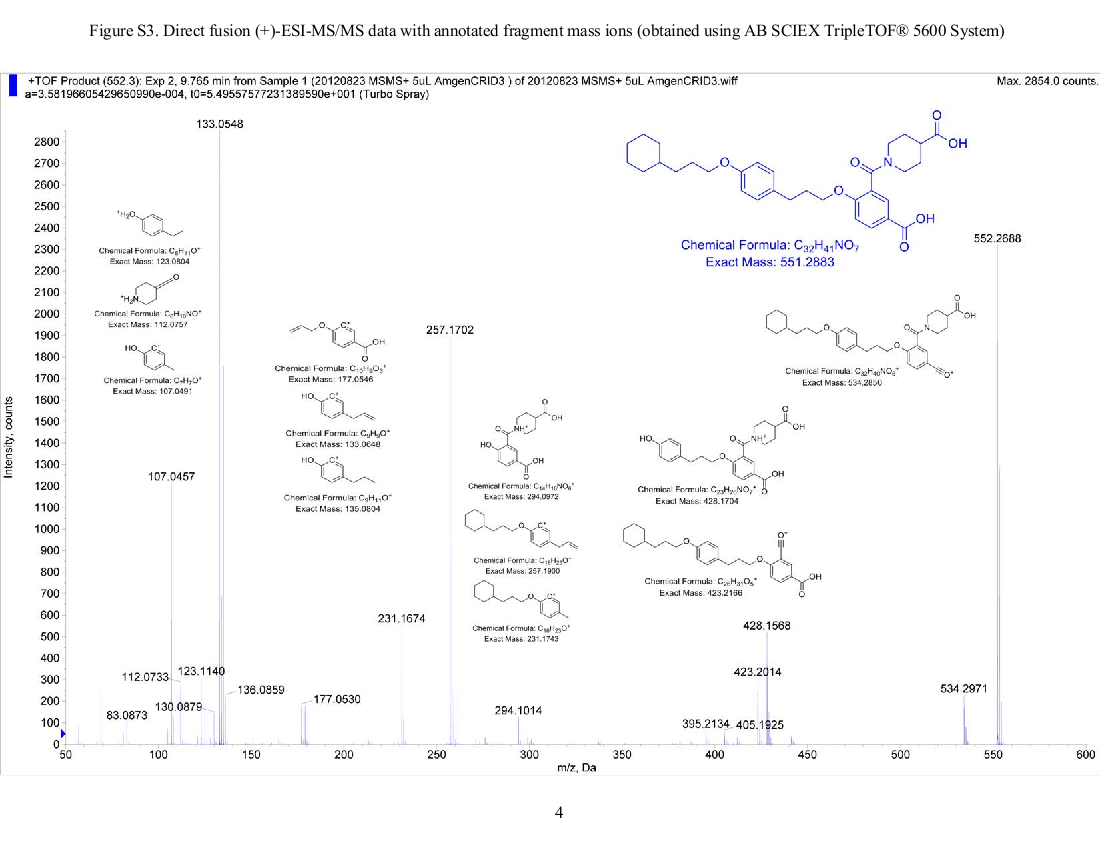

Supplement: Supplementary file 4 [file pone.9f221489-155d-4978-a36d-30c51853e438.s004.tif]

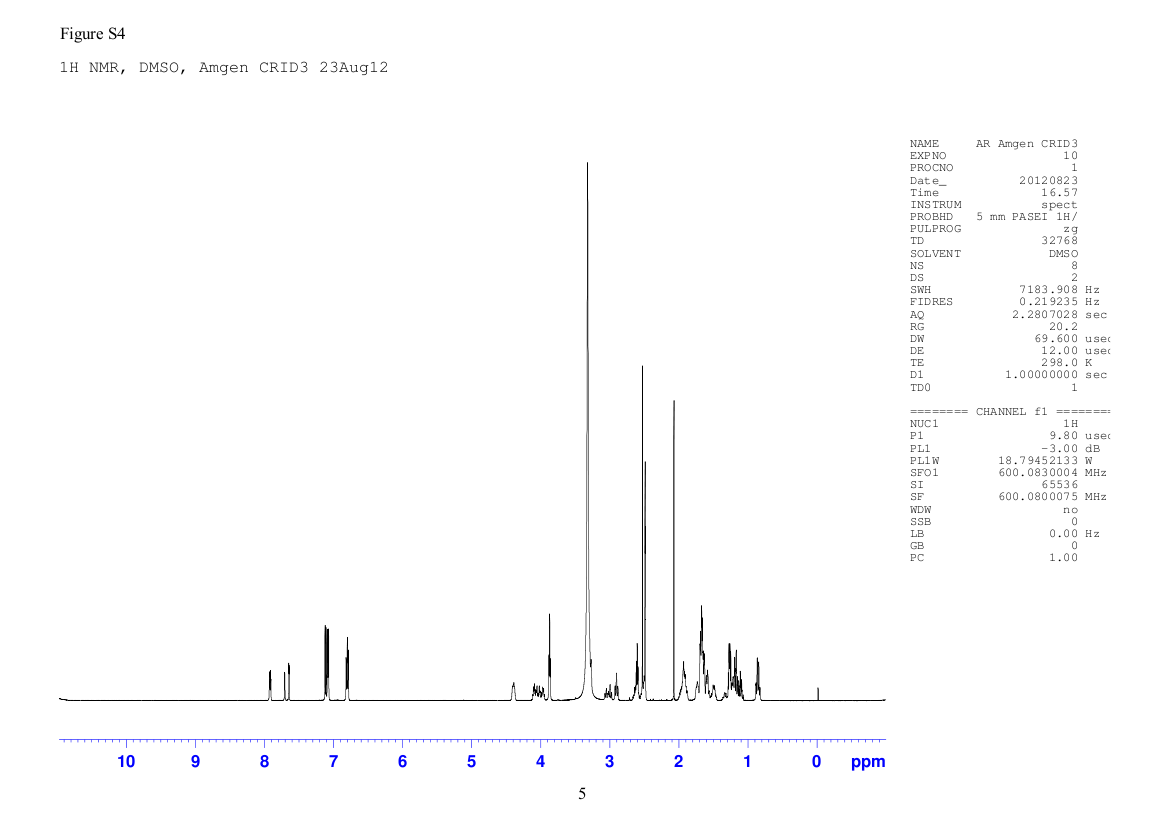

Supplement: Supplementary file 5 [file pone.9f221489-155d-4978-a36d-30c51853e438.s005.tif]

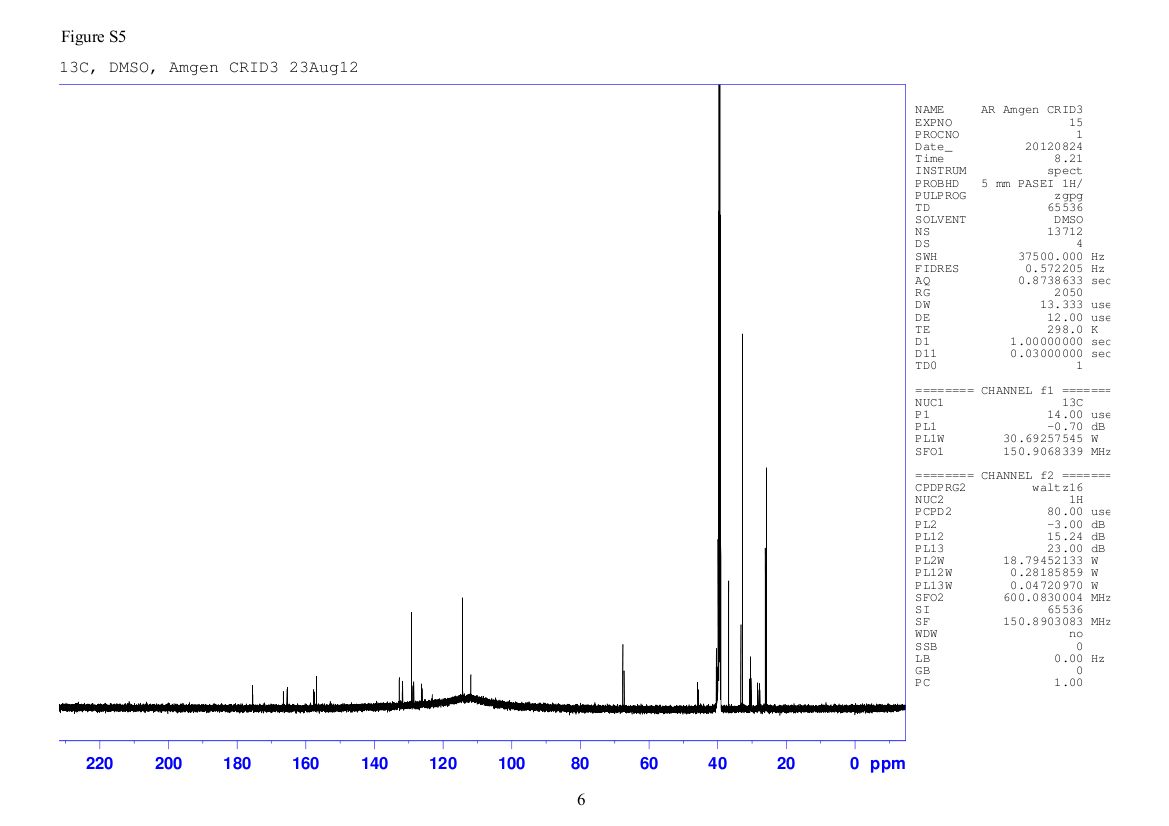

Supplement: Supplementary file 6 [file pone.9f221489-155d-4978-a36d-30c51853e438.s006.tif]

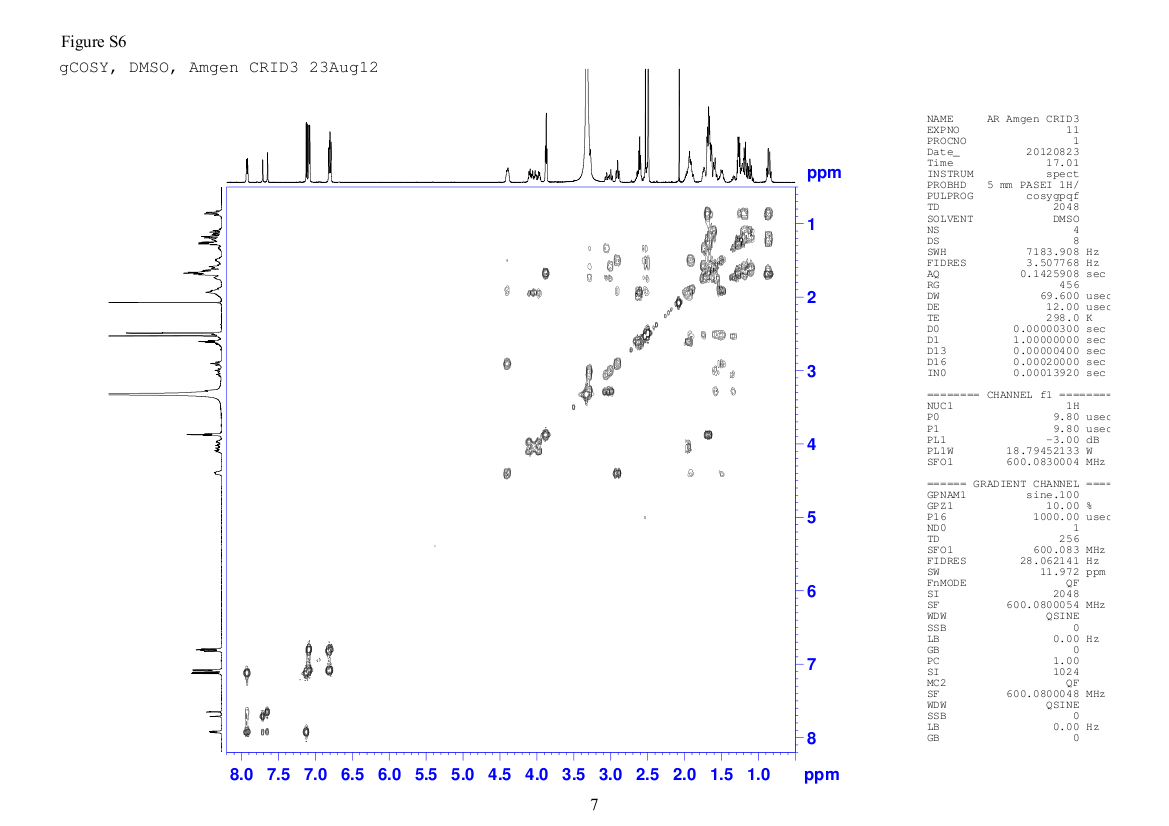

Supplement: Supplementary file 7 [file pone.9f221489-155d-4978-a36d-30c51853e438.s007.tif]

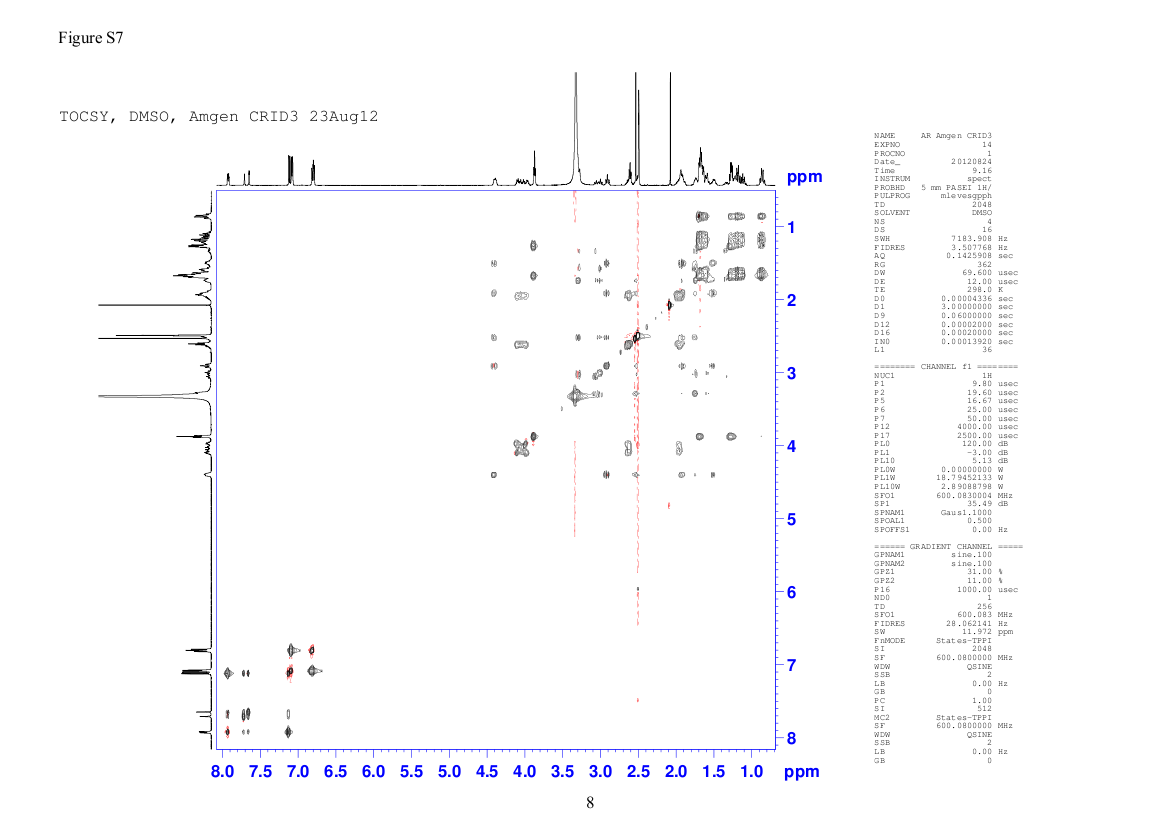

Supplement: Supplementary file 8 [file pone.9f221489-155d-4978-a36d-30c51853e438.s008.tif]

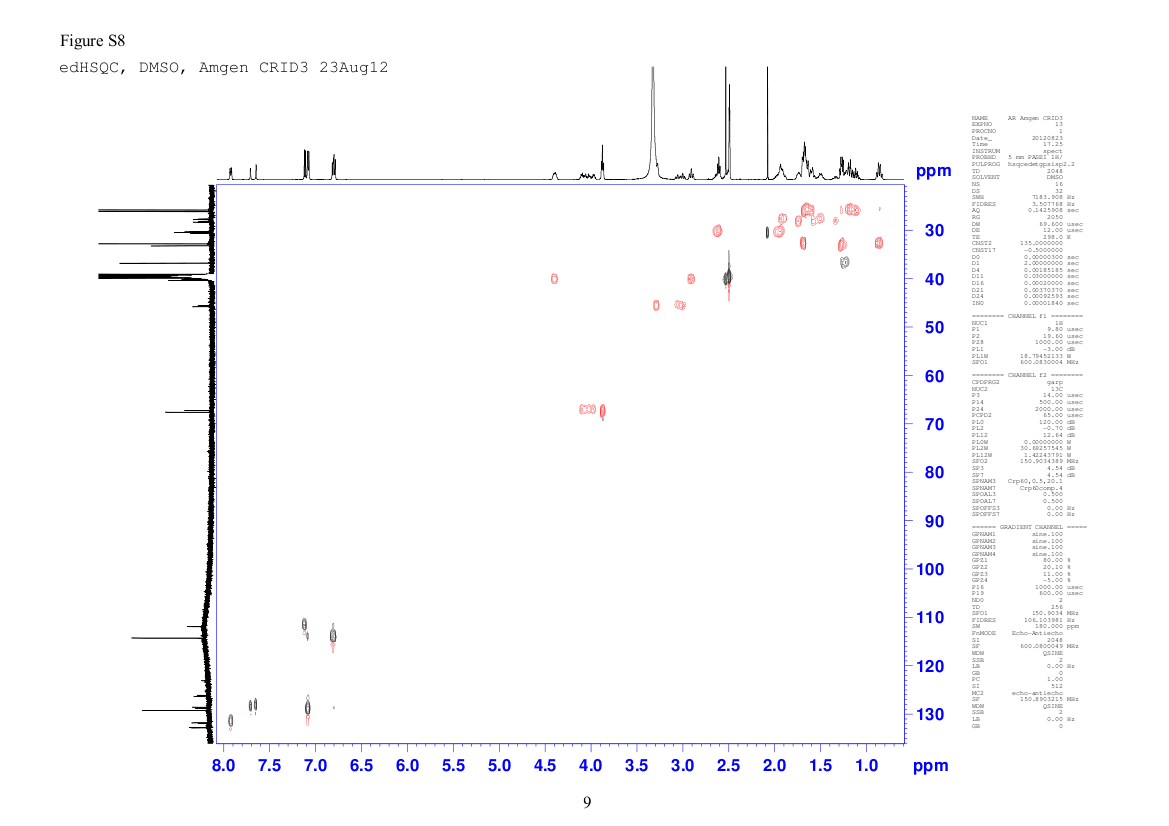

Supplement: Supplementary file 9 [file pone.9f221489-155d-4978-a36d-30c51853e438.s009.tif]

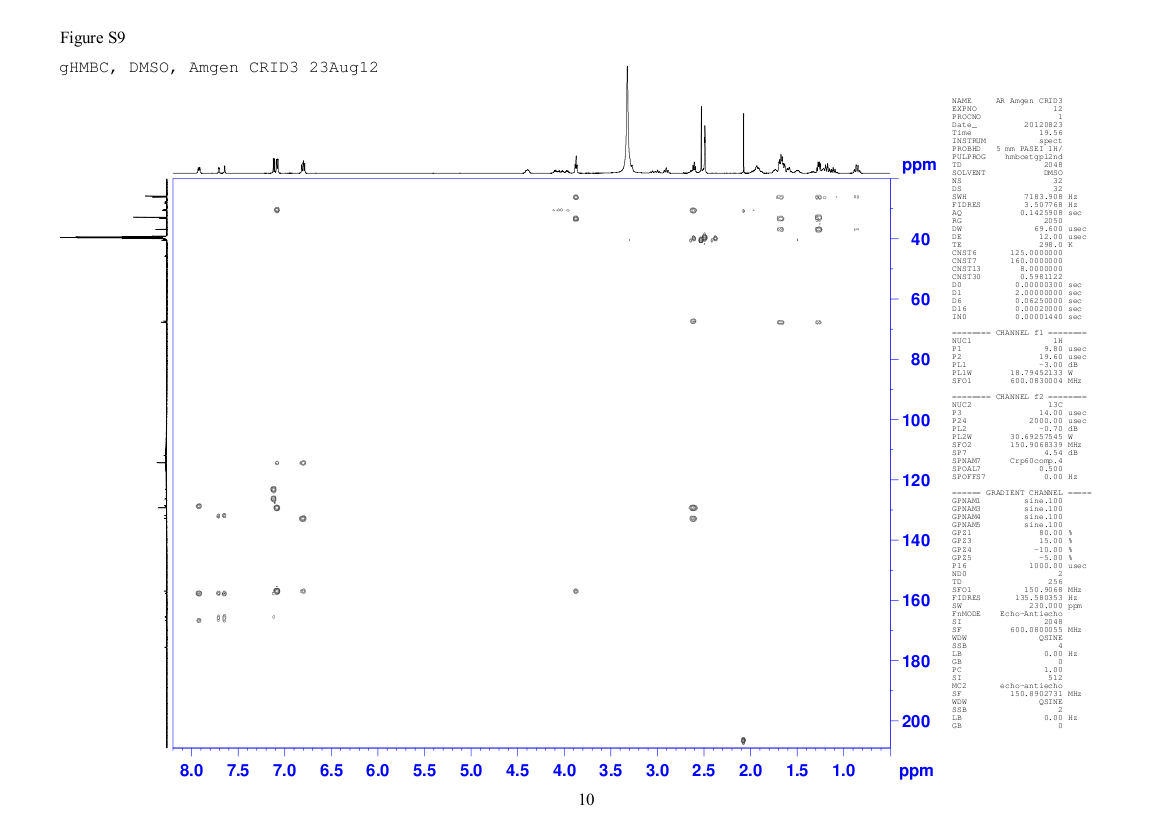

Supplement: Supplementary file 10 [file pone.9f221489-155d-4978-a36d-30c51853e438.s0010.tif]
